# Implementation of evidence-based antenatal care in Mozambique: a cluster randomized controlled trial: study protocol

**DOI:** 10.1186/1472-6963-14-228

**Published:** 2014-05-21

**Authors:** Leonardo Chavane, Mario Merialdi, Ana Pilar Betrán, Jennifer Requejo-Harris, Eduardo Bergel, Alicia Aleman, Mercedes Colomar, Maria Luisa Cafferata, Alicia Carbonell, Beatrice Crahay, Therese Delvaux, Diederike Geelhoed, Metin Gülmezoglu, Celsa Regina Malapende, Armando Melo, My Huong Nguyen, Nafissa Bique Osman, Mariana Widmer, Marleen Temmerman, Fernando Althabe

**Affiliations:** 1 Mozambique Ministry of Health, Av. Eduardo Mondlane/Salvador Allende Nº 1008, Bairro Central, C.P. 264 Maputo, Mozambique; 2 Department of Reproductive Health Research, World Health Organization, Geneva, Switzerland; 3 Institute for International Programs, Johns Hopkins Bloomberg School of Public Health, Baltimore, Maryland, USA; 4 Institute for Clinical Effectiveness and Health Policy (IECS), Dr. Emilio Ravignani 2024, C1414CPV Buenos Aires, Argentina; 5 Montevideo Clinical and Epidemiological Research Unit (UNICEM), Montevideo, Uruguay; 6 Mozambique Country Office, World Health Organization, Maputo, Mozambique; 7 International Center for Reproductive Health (ICRH-M), Avenida Maquiguana 100, Maputo, Mozambique; 8 Institute of Tropical Medicine, Antwerp, Belgium; 9 Central de Medicamentos e Artigos Médicos, Maputo, Mozambique

**Keywords:** Antenatal care, Pregnancy

## Abstract

**Background:**

Antenatal care (ANC) reduces maternal and perinatal morbidity and mortality directly through the detection and treatment of pregnancy-related illnesses, and indirectly through the detection of women at increased risk of delivery complications. The potential benefits of quality antenatal care services are most significant in low-resource countries where morbidity and mortality levels among women of reproductive age and neonates are higher.

WHO developed an ANC model that recommended the delivery of services scientifically proven to improve maternal, perinatal and neonatal outcomes. The aim of this study is to determine the effect of an intervention designed to increase the use of the package of evidence-based services included in the WHO ANC model in Mozambique. The primary hypothesis is that the intervention will increase the use of evidence-based practices during ANC visits in comparison to the standard dissemination channels currently used in the country.

**Methods:**

This is a demonstration project to be developed through a facility-based cluster randomized controlled trial with a stepped wedge design. The intervention was tailored, based on formative research findings, to be readily applicable to local prenatal care services and acceptable to local pregnant women and health providers. The intervention includes four components: the provision of kits with all necessary medicines and laboratory supplies for ANC (medical and non-medical equipment), a storage system, a tracking system, and training sessions for health care providers. Ten clinics were selected and will start receiving the intervention in a random order. Outcomes will be computed at each time point when a new clinic starts the intervention. The primary outcomes are the delivery of selected health care practices to women attending the first ANC visit, and secondary outcomes are the delivery of selected health care practices to women attending second and higher ANC visits as well as the attitude of midwives in relation to adopting the practices. This demonstration project is pragmatic in orientation and will be conducted under routine conditions.

**Discussion:**

There is an urgent need for effective and sustainable scaling-up approaches of health interventions in low-resource countries. This can only be accomplished by the engagement of the country’s health stakeholders at all levels. This project aims to achieve improvement in the quality of antenatal care in Mozambique through the implementation of a multifaceted intervention on three levels: policy, organizational and health care delivery levels. The implementation of the trial will probably require a change in accountability and behaviour of health care providers and we expect this change in ‘habits’ will contribute to obtaining reliable health indicators, not only related to research issues, but also to health care outcomes derived from the new health care model. At policy level, the results of this study may suggest a need for revision of the supply chain management system. Given that supply chain management is a major challenge for many low-resource countries, we envisage that important lessons on how to improve the supply chain in Mozambique and other similar settings, will be drawn from this study.

**Trial registration:**

Pan African Clinical Trial Registry database. Identification number: PACTR201306000550192.

## Background

### Antenatal care (ANC) and MDGs

In 2000, the United Nations Millennium Declaration and 8 associated Millennium Development Goals (MDGs) were adopted by 189 countries, signifying the establishment of an unprecedented global partnership to meet the needs of the world’s poorest populations [1]. MDG 4 calls for a two-third reduction of infant mortality by 2015, and MDG 5 calls for a 75% reduction of 1990 maternal mortality levels by 2015 (MDG 5A), and universal access to reproductive health (MDG 5B) [2,3]. Antenatal care (ANC) indicators, both at least one visit and four or more (4+) visits, are MDG 5B tracking indicators – a recognition in the international community of the central importance of ANC to women's health.

ANC reduces maternal and perinatal morbidity and mortality directly through the detection and treatment of pregnancy-related illnesses, and indirectly through the detection of women at increased risk of delivery complications [4,5]. Studies showing an association between ANC utilization and uptake of skilled delivery care also suggest that ANC can save women and newborn lives through the promotion of skilled delivery care and counseling on birth planning and complication readiness [6-8].

ANC visits constitute one of the few times women in many resource-poor settings seek care for their own health [9,10] and, therefore, represent an important opportunity for: 1) reaching women with a number of interventions that may be vital for their health and the health of their unborn child (e.g. anaemia, HIV, syphilis and malaria prevention, detection and treatment), and 2) informing women about pregnancy, related complications, and the advantages of skilled delivery care as well as the importance of birth spacing [11,12].

### Current status of ANC in high burden countries

The potential benefits of quality ANC services are most significant in low-resource countries where morbidity and mortality levels among women of reproductive age and neonates are, on average, highest. The Countdown 2013 report which tracks progress in the 75 countries in the world with the greatest burden of maternal, newborn, and child mortality where over 95% of all maternal and child deaths occur, shows a median coverage level across these countries of 57% for ANC four or more visits (range in coverage is 15% to 94%) [13].

These figures show that although the delivery and prioritization of ANC differs across the 75 Countdown countries, there is an overall need to increase antenatal care attendance by women. Mozambique, a priority country for Countdown, is not an exception. According to the Mozambique Demographic and Health Survey (DHS) 2011, 91% of women received at least one ANC visit (up from 71% in the 1997 DHS), while 51% received 4 or more visits, with coverage being higher in urban compared with rural areas (60% vs 47%). The median gestational age at the first ANC visit was 4–5 months with no differences between urban or rural areas [14].

### WHO ANC model

From 1996–1998, the Department of Reproductive Health and Research (RHR) at the World Health Organization (WHO) conducted a multicentre cluster-randomized controlled trial to evaluate a new ANC model that recommended the delivery of services scientifically proven to improve maternal, perinatal and neonatal outcomes through four antenatal and one postpartum visit (for women with no evidence of complications). This new ANC care model was assessed against local models which required substantially more ANC visits. The WHO model divides pregnant women into two groups: 1) those with no evidence of pregnancy-related complications, medical conditions or major health-related risk factors (75% of the total population of pregnant women) and eligible for the basic package of ANC services, and 2) those with specific health conditions or risk factors that necessitate special care (25% of pregnant women). The trial found that the new WHO model produced maternal and perinatal outcomes comparable to the local models, and was readily accepted by women and health care providers [15].

A subsequent WHO systematic review of randomized controlled trials of routine ANC similarly concluded that a model with a reduced number of antenatal visits could be introduced into clinical practice without risk to mother or baby [16].

### Implementation of the WHO ANC model

The WHO ANC model has been implemented in Thailand, Argentina and Micronesia with positives results. In anticipation of the potential difficulties of translating research findings on ANC into clinical practice, Thailand developed an implementation plan for the new WHO ANC model before the 1996–1998 trial started. This plan clearly defined the coordinated actions to be taken at all levels of the health-care system, from consumers, health professionals, responsible country authorities to international organizations, for successful introduction of the model [17].

In Argentina, policy-makers at national and local levels were involved in rolling out the WHO ANC model after its implementation was approved in early 2003 (unpublished information, personal communication, Centro Rosarino de Estudios Perinatales, CREP). The WHO ANC model has been fully institutionalized throughout Yap State in the Federated States of Micronesia (unpublished information, personal communication, Centro Rosarino de Estudios Perinatales, CREP). In all cases, implementing the new model required coordinated efforts among all actors involved in the ANC process, training sessions to the personnel (in person or on line) and dissemination strategies to make the ANC model familiar to health-care workers.

The above are good examples of how research findings on ANC can be effectively translated into clinical practice. However, the implementation processes adopted in the three countries were not carried out within the framework of a rigorous research protocol. Consequently, there is no strong evidence showing that the adopted strategies are significantly better than other approaches to implementing the WHO ANC model. It is also not clear whether the strategies can be replicated in other similar contexts, as they were designed in relation to specific local conditions in the three countries. This proposed study will address this knowledge gap by generating new evidence based on the findings of a methodologically sound intervention protocol.

### Mozambique ANC model

In December 2008, the Ministry of Health of Mozambique launched the *Integrated Plan for the achievement of MDGs 4 and 5*. The plan included an integrated package of interventions for pregnant women, newborns and children below 5 years of age. The interventions for pregnant women are based on the WHO ANC model in a minimum of four antenatal visits and one post-partum visit including basic interventions for non-complicated pregnancies and additional interventions for complicated pregnancies.

The initial experience with the introduction of the ANC model in Mozambique has indicated that bottlenecks and health system deficiencies are limiting the full implementation of the model at health facility level. A meeting of experts convened in Maputo in 2009 by the Ministry of Health, the WHO, and the Flanders International Cooperation Agency (FICA) highlighted lack of motivation and knowledge among care providers as potential determinants of this situation.

This proposed study builds extensively on the recommendations of this meeting and is considered by the Ministry of Health as an important opportunity to generate knowledge for the development of strategies to improve the quality and integration of ANC services at national level. All references to the “ANC model” in the remainder of this protocol concern the Mozambique ANC model.

### Evidence-based strategies to disseminate and implement maternal and child health services at primary care level

A major challenge in developing countries is to bridge the ‘know-do gap’ and make scientifically proven interventions widely available to underserved populations. There is an urgent need for new approaches that will enable scaling-up of effective and beneficial interventions in developing country contexts. Evidence shows that quality improvement interventions (broadly defined as strategies to ensure delivery of effective services efficiently and equitably) can be used to disseminate evidence-based practices among clinicians in high income countries [18-20]. Whether this can be achieved in resource-poor settings is uncertain, as there is little research done in low or middle-income countries (LMIC).

A systematic review of systematic reviews published in 2008 analyzed strategies for improving the quality of care of maternal and child health (MCH) services in developing countries [21]. The 23 systematic reviews (including 9 Cochrane reviews) evaluated the effects of continuing education and quality improvement strategies, financial and reimbursement strategies (e.g. provider incentives), and organization of care strategies on the delivery of MCH services. The main findings of the overview can be summarized as follows: 1) Interactive workshops, reminders and multifaceted interventions (that combine two or more strategies) can improve health care practice, and generally have moderate effects; 2) Educational outreach visits consistently improve targeted health seeking behaviours but have variable effects on other behaviours contributing to poor health outcomes; 3) Audit, feedback and the use of opinion leader interventions have small to moderate effects on provider practices. There was not enough information at that time to draw implications for clinical practice or public health policy from other quality improvement strategies including financial incentives, and organizational or regulatory interventions.

One key study testing a strategy for implementing evidence-based childbirth and other maternal care practices in developing countries was conducted in Latin America during 2004–2007. This was a multicenter cluster randomized trial in 19 public maternity hospitals in Argentina and Uruguay with the specific aim to increase the use of two evidence-based practices during childbirth: the use of oxytocin during the third stage of labor and selective episiotomy. The trial evaluated the effect of a behavioural intervention on the implementation of evidence-based clinical guidelines on the prevention of post-partum haemorrhage and the use of episiotomy. A multifaceted approach consisting of training to perform evidence-based practices, academic detailing, reminders and feedback was used to disseminate, implement, and maintain the guidelines in the hospitals. This multifaceted approach showed changes and sustainability in the practices under study [22].

### Study aims and hypotheses

The ***main specific aim*** of this study is to determine the effect of an intervention designed to increase the use of evidence-based practices included in the ANC package by midwives (and other health professionals) in prenatal clinics in Mozambique. Specifically, we will assess the effect of the intervention on practices related to the detection, treatment and prevention of major health-related conditions (e.g., anaemia, and infectious diseases such as HIV/AIDS, malaria, and congenital syphilis).

Our ***primary hypothesis*** is that the intervention will increase the use of such practices in comparison to the standard dissemination channels currently used in the country. A secondary hypothesis is that the intervention will improve the attitudes and readiness of midwives (and other health professionals) to provide all recommended practices during ANC visits to their women clients.

We set out to assess if a multifaceted intervention with several interacting components to increase the use of evidence-based practices in an ANC package would be effective in improving the quality of care of maternal and child health services in Mozambique [19].

## Methods

**Figure 1:**
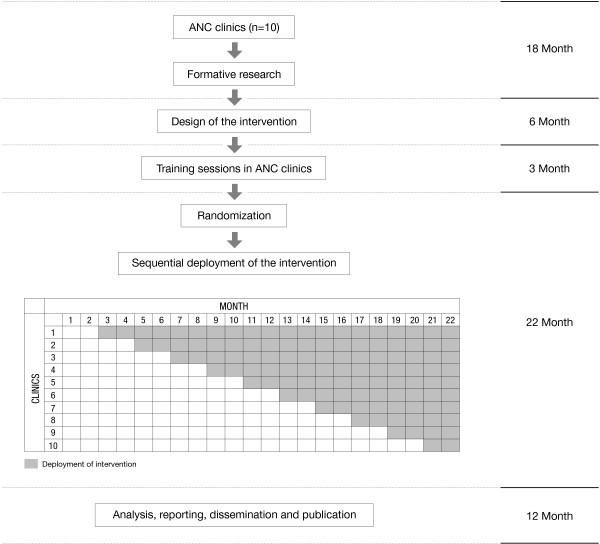
Flow chart of the trial.

This will be a facility-based cluster randomized controlled trial with a stepped wedge design. Figure 1 summarizes the study design. The intervention will be rolled out sequentially to ten ANC clinics in three regions of Mozambique starting a new facility every two months. After two months of baseline data collection in all facilities (months 1 and 2), facility 1 will start at month 3, facility 2 will start at month 5, etc. The order in which the clinics receive the intervention will be determined at random and, by the end of study, all clinics will have received the intervention. Outcomes will be computed at each time point when a new clinic starts the intervention.

Complex interventions such as that planned for this trial are challenging to evaluate because of the organizational and logistical difficulties involved in designing and delivering the intervention in a standardized way across participating facilities. Interventions aimed at improving quality of care and access to services are also often ethically and politically challenging to withhold from a proportion of participant clusters (or to withdraw the intervention as would occur in a cross-over design) [23]. In addition, because of logistical and financial constraints, the researchers determined that the planned intervention could only be implemented in stages. In such circumstances, determining the order in which clusters receive the intervention at random is more acceptable to implementers and policy makers. For all of these reasons, the stepped wedge design was considered advantageous to use for this trial [24].

### Study population

#### ***Participating clusters and women***

**Figure 2:**
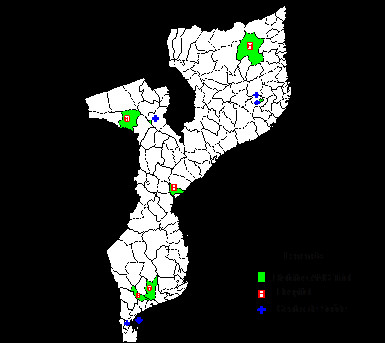
**Map of Mozambique showing the regions and pre-selected ANC clinics.** Credits: Siv Steffen Nygaard, WHO Mozambique.

This study will be conducted in 10 ANC clinics in the three regions of Mozambique. The Ministry of Health pre-selected the 10 clinics according to their programmatic activities and priorities. The pre-selected clinics are shown in Table 1 and Figure 2. They account for a significant proportion of the annual ANC visits in the country.

**Table 1:** Pre-selected ANC clinics

**Region**	**Health Center (HC)**	**Women attending 1**^**st **^**ANC in 2011**
**South**		
**Maputo City**	HC 1ro de Maio	3,165
**Maputo Province**	HC da Matola II	3,276
**Gaza**	HC annexed to the rural hospital of Chowke	3,480
**Gaza**	HC of Chibuto	9,756
**Central**		
**Sofala**	HC annexed to the district hospital of Dondo	2,728
**Tete**	HC annexed to the rural hospital of Songo	1281
**Tete City**	HC N° 2	2778
**North**		
**Nampula City**	HC 25 de Setembro	5,960
**Nampula District**	HC of Anchilo	1,894
**Cabo Delgado**	HC annexed to the rural hospital of Montepuez	4,182

Clinics were eligible if (1) they were not already implementing the proposed ANC model; (2) they served at least 200 new pregnant women per year; (3) they had midwives or nurses-midwives among their personnel; and (4) they were willing to participate. All women attending ANC visits at the participating clinics will be eligible to receive the ANC package.

#### ***Sample size calculation***

The sample size was estimated very conservatively, assuming a baseline frequency of selected health practices of 30%, and an increase to 60% in the prenatal clinics assigned to the intervention group. For a 0.05 alpha level, 80% power and an intra-cluster correlation coefficient of 0.05, six clusters will be needed. To protect against pre-randomization exclusions and drop outs, 10 clinics will be included.

### The intervention

The intervention was designed and tailored based on the results of an analysis of previously collected data by the Mozambique Ministry of Health from the National Needs Assessment on Maternal and Neonatal Health [25] and the results of formative research using qualitative methods.

#### ***National needs assessment on maternal and neonatal health***

The Ministry of Health of Mozambique conducted a national survey at facility level to assess the quality of care of maternal and child health services [25]. The survey was conducted in facilities selected randomly and consisted of several modules focusing on human resources available, characteristics of prenatal care, women’s satisfaction, maternal and neonatal health indicators, and information on barriers and factors that contribute to the delay in seeking appropriate medical help for obstetric care.

In the context of this trial, the data from the survey were analyzed for the facilities that had been pre-selected for this demonstration project by the Ministry of Health to provide information on the current rates of use of the evidence-based practices included in the ANC package, which practices were underutilized or not utilized and what were the barriers and facilitating factors to the correct implementation of the selected practices. Five of the pre-selected facilities were included in the survey and all of them have antenatal care services.

#### ***Formative research***

Understanding the barriers that hinder health practitioners’ ability or willingness to implement evidence based practices is critical for the implementation of any strategy aimed at improving provider uptake of these practices [26]. Thus, a qualitative study was carried out in 2011 during the preparatory phase of the trial to ensure that the implementation strategy would be effective, culturally appropriate, and accepted by women and ANC providers [27,28].

The specific aims were:

1. To obtain and analyze information to define the components of the intervention strategy by: a) Identifying system and individual factors which influence the successful adoption of scientific recommendations in obstetric practice, and mechanisms for effectively introducing evidence-based practices within routine prenatal care; and b) Identifying the barriers and facilitating factors for the implementation of the evidence-based specific practices in the WHO ANC package at prenatal clinics.

2. To evaluate the appropriateness of the proposed intervention by: a) Assessing prenatal care providers’ and clinic authorities’ perspectives regarding the components of the intervention; b) Assessing women’s perspectives on routine ANC.

The methods used included focus groups with prenatal care providers and women, and in-depth interviews with clinics authorities as needed. The main barriers identified regarding the service and behavioral components were the lack of infrastructure, instruments and materials, and stock of products needed for delivery of recommended ANC services, inappropriate training of the personnel involved in all stages of ANC, and the lack of a registration system for records. Regarding the procurement and supply chain management component, barriers were related to qualified stock records, reliable data and weak implementation of a supply system including transport.

#### ***Description of the intervention***

The multifaceted intervention includes the following four components:

**Table 2:** Types of kits and content to be produced and used at the clinics

**Kit**	**Type of visit**	**Overall content**	**Specific content**
**A**	First visit	All products needed for the 1^st^ consultation	Test strips for urine proteins, hemoglobin, Rapid Diagnostic Test for HIV and syphilis, benzathine penicillin, mebendazole, sufadoxine-pyrimethamanine, ferrous sulphate/folic acid tablets, cotrimoxazole, antiretroviral (ARV) as well as medical devices to administer these medications and perform clinical examinations.
**B**	Follow up visit	All products needed for the follow up consultations	The same components of kit A except for syphilis rapid test, benzathine penicillin and mebendazole.
**C**	First and follow up visits	Urine collecting containers	
**D**	First and follow up visit	Long lasting impregnated bed nets	

COMPONENT 1: ANC kits containing the necessary medicines, laboratory supplies, materials and equipment.

Four different kits were designed. Each clinic will be provided with the ANC kits which include the products required for the ANC consultations (first and follow-up visits) according to the current protocols and norms legislated by the Ministry of Health. The number of kits has been calculated and designed so that each clinic will have provisions for one month. Table 2 shows the 4 types of kits that will be produced and used at the clinics.

COMPONENT 2: Availability of a cupboard

There will be a cupboard in the room where the ANC visit will take place, which will serve as a storage place for the kits. This will allow easy and quick access of the nurses to all necessary materials while the woman is in the room, enabling the nurses to provide care comprehensively and improving the delivery and quality of the necessary ANC interventions in a most efficient manner.

COMPONENT 3: Tracking sheet

The intervention includes the use of a tracking sheet (so-called “ficha”) for the research assistants to monitor the stock levels of the various products in the kits. The tracking sheet will enable the study to monitor kit use and avoid stock-outs.

COMPONENT 4: Training session

At the start of the intervention in each site, a refresher course on the essential and complementary interventions for ANC will be given to all the MCH nurses working in the health facilities of the study. MCH nurses are responsible for delivering ANC in Mozambique. They will also be re-trained in using the materials and products, such as proteinuria test, blood pressure measurement, etc. The training will also include the use of the kits, use of the tracking sheet and procedures to ensure continuous supply of the medicines and materials needed. The training session will be a 3-day course and it will be led by the Ministry of Health in collaboration with the partners involved in this project.

In accordance with the stepped wedge design, the intervention will be rolled out sequentially to the ten ANC clinics in an order that will be decided randomly. Before the intervention is rolled out in a particular clinic, this clinic will receive no intervention but will be asked to continue with standard in-service training activities.

This demonstration project is pragmatic in orientation and will be conducted under routine conditions. Under such conditions, non-compliance is embedded in the evaluation. Reasonable measures will be implemented to improve compliance [29].

### Outcome measures

#### ***Primary outcomes***

The primary outcome will be the delivery of selected health care practices to women attending the first antenatal care visit. Three practices will be chosen from the list of priority practices below, after evaluating their current use during the baseline period and before implementation of the intervention. Practices that are not selected as primary outcomes will be incorporated as secondary outcomes.

• Frequency of women receiving screening for syphilis

• Frequency of women receiving screening for HIV

• Frequency of women receiving screening for anaemia

• Frequency of women receiving screening for hypertension

• Frequency of women receiving tetanus toxoid

• Frequency of women receiving intermittent preventive malaria treatment

• Frequency of women receiving iron supplementation

• Frequency of women receiving anti-parasitic treatment (de-worming)

• Frequency of syphilis sero-positive women receiving the administration of syphilis treatment

• Frequency of HIV sero-positive women receiving administration of antiretroviral treatment.

#### ***Secondary outcomes***

Selected practices in each ANC visit after the first ANC visit:

• Frequency of women receiving screening for syphilis

• Frequency of women receiving screening for HIV

• Frequency of women receiving screening for anaemia

• Frequency of women receiving screening for hypertension

• Frequency of women receiving tetanus toxoid

• Frequency of women receiving intermittent preventive malaria treatment

• Frequency of women receiving iron supplementation

• Frequency of women receiving anti-parasitic treatment (de-worming)

• Frequency of syphilis sero-positive women receiving the administration of syphilis treatment

• Frequency of HIV sero-positive women receiving administration of antiretroviral treatment

• Frequency of midwives’ positives attitudes and readiness to provide the selected practices.

#### ***Composite outcome***

A score will be computed. One point will be added to the score for each selected practice that was implemented. A woman receiving all selected practices will get the highest possible score, while a woman who receives none of the practices will get a score equal to zero. Two sets of scores will be computed, one for the first ANC visit, and one for subsequent visits.

### Process measures

Process data will be collected during the intervention period at prenatal clinics. The objectives of this process evaluation are: 1) to detect implementation problems that could be explanatory in case the intervention was not effective; and 2) to facilitate the replication and scale-up of the intervention if it is proven effective.

### Data collection and management

#### ***Source of data for primary outcomes***

The Ministry of Health of Mozambique has designed a data collection instrument to be used for the registration of ANC data at national level in order to obtain uniform and comprehensive data from all health units. This pre-printed Book of Registration of Antenatal Care Data (Livro de Registro da Consulta Pre-natal) has been distributed to all health facilities, and all ANC providers are required to complete it for each ANC visit. The unit of registration is the antenatal visit (not the woman visited). However, in order to be able to compute individual-based indicators, a system will be in place by which each woman will be assigned a code/number that will be reflected in the logbook. The visits and the women will be linked by this code maintaining, however, the anonymity of the woman. This will be achieved by means of a card (the size of a visiting card) that would be given to the mother on the first ANC visit and should be carried with her ANC card to every ANC visit. This card will be assigned a code/number and will be composed of 12–15 small stickers with the same code/number. When the woman comes to the ANC visit, the nurse will take one of the stickers and place it in the logbook in the row corresponding to that visit.

The variables in the registration book are the following: date of prenatal visit, name, age, address, number of ANC visits for this pregnancy, gestational age, blood pressure, malnutrition, anaemia, proteinuria, examination of the breast (presence of nodules), screening and treatment of syphilis (for the woman and her partner) and other sexually transmitted infections, tetanus immunization status, screening, prevention and treatment of maternal-to-child transmission of HIV, prevention of malaria with intermittent preventive treatment, iron administration, anti-parasitic treatment, distribution of bed nets, partner coming to the visit, reference to another health facility and name of health provider.

#### ***Quality data monitoring and quality control***

A comprehensive quality assurance and quality control will be put in place to ensure that the best possible data will be filled in the registration book and entered into the database. Guidelines for collecting data and filling in the registration book will be produced. To ensure that the data will be collected in a standardized way in all the participating clinics, a pilot testing of data collection and data management will be conducted prior to the start of the baseline data collection. Data quality monitoring reports will be produced regularly for each clinic. Site monitoring visits to the participating clinics will be conducted routinely and source data verification (SDV) will be performed to ensure that the data collected are complete, accurate and reliable.

Additionally, data quality control mechanisms will be implemented in order to ensure reliability of the data obtained through routine data collection. These mechanisms will include surveys on a random sample of pregnant women attending ANC clinics at exit from the facilities. Selected women will be approached as they are leaving the facility after the ANC visit to conduct the exit survey and they will be asked to participate by signing a consent form. These women will be invited to answer a set of questions related to the practices and other variables collected in the registration book (primary source of data) in order to evaluate if the information registered is identical to the data collected in the exit survey. If differences are observed, providers will be interviewed in order to resolve them. Three surveys will be conducted for this purpose at three points in time; each time point with 1000 surveys (100 questionnaires per clinic). The first survey will be carried out just before starting intervention; the second at 12 months and the last at 20 months, approximately.

#### ***Source of data for secondary outcomes: willingness of health professionals who provide antenatal care to adopt the practices recommended in the WHO model***

The data will be collected at two data points: one within the first four months of the study period and the second within the last two months of the study period. A self-administered questionnaire on paper forms will be used. These forms will be sent to the data management center.

This survey is designed to assess the willingness of health professionals who provide antenatal care to adopt the practices recommended. The questions are about their perceptions of these practices and viewpoints on any barriers or facilitating factors that may affect the adoption of these practices and national guidelines.

The survey will be administered in all participating health care centers. The information obtained from this questionnaire the first time will be used as baseline data and will be compared to results from the same questionnaire administered after the intervention. The project data collector will be responsible for the administration of the surveys, and informed consent will be obtained from all participants. The questionnaire will be provided to health care professionals in an unmarked opaque envelope.

#### ***Data management***

A web-based data management (DM) system will be used to enter the data and to monitor the data quality. The system will be developed by the Biostatistics and Data Management (SIS) team at UNDP/UNFPA/UNICEF/WHO/World Bank Special Programme of Research, Development and Research Training in Human Reproduction (HRP) at WHO in Geneva using the OpenClinica Application available at: https://www.openclinica.com, version 3.1.3 [30]. The system will include programmed edit checks to validate completeness, accuracy and consistency of the data entered. The OpenClinica system is an open-source software, highly secure and reliable web-based solution, fully compliant with Good Clinical Practice (GCP) and regulatory guidelines as well as the HRP/WHO Standard Operating Procedures (SOPs) for managing clinical trials. These procedures have been used in previous international multicentre trials sponsored or coordinated by WHO in the past five years and proven to be efficient. The online data entry system minimizes the delays in data queries, completion of forms and problem resolution; it makes the dataset quickly available and permits continuous assessment of data quality.

Data management will be performed locally in Maputo by the Consultorio de Estatistica e Serviço de Soluçoes-LDA (CESS) and by the WHO/SIS team. The team in Maputo will be trained in data-entry and data management using the OpenClinica system.

The nurses of all participating clinics will be trained in data collection and filling the registration book following an instructions booklet. Research assistants from clinics will be trained to take digital photos of the registration book and send them to the data management center. Photos will be sent from each facility by email on weekly basis. CESS will be responsible for verifying the data from the photos, uploading them to *dropbox* and entering the data into the OpenClinica system. Double data entry will be performed to minimize errors. Queries on data (missing values, outliers, inconsistencies and other errors) will be generated automatically in the system. Data quality will be monitored online by the WHO/SIS Team at WHO.

Standard ASCII or SPSS or SAS datasets will be generated from the final study database ready for analysis.

### Statistical analysis

Descriptive statistics will be reported by computing frequencies and percentages for categorical variables and means, standard deviations, and minimum and maximum values for continuous variables. Distribution of the variables will be examined to detect the outliers as a part of quality control and descriptive analysis of the data. Descriptive statistics will be tabulated for individual clusters and aggregated across clusters.

There have been many suggested approaches to the analysis of stepped wedge designs, ranging from repeated application of purely between-unit analysis for each step, to the application of purely within-unit analysis, usually performed by some form of interrupted time series analysis. We will follow the general approach set out by Hussey *et al.*[31].

The primary objective of the study is to estimate the effectiveness of the intervention. The generalised linear mixed model (GLMM) will be used in this analysis to provide an estimate of the intervention effect, using both between and within ANC clinics effects. STATA and SAS statistical packages will be used for the analysis.

### Duration of the project

The proposed study will require 61 months including the formative research. The deployment of the intervention will required 22 months (See Figure 1).

### Limitations

There are some limitations regarding the stepped weighed design, such as a greater complexity in the implementation of the intervention, in some case a longer trial duration. However the strengths outweigh the limitations.

### Ethical aspects

This protocol has been approved by the Research Project Review Panel of the UNDP/UNFPA/UNICEF/WHO/World Bank Special Programme of Research, Development and Research Training in Human Reproduction at the Department of Reproductive Health and Research of WHO, and the WHO Research Ethics Review Committee, Geneva, Switzerland. The Comité Nacional de Bioéticapara a Saúde of the Ministry of Health in Mozambique also approved the study.

A waiver of consent forms for participating women was obtained since this study is considered of minimal risk (risks of daily life, and includes the risks associated with routine physical examinations and review of medical records) and it is developed in the context of regular prenatal care and using as research records those used in health care process (no data collection tools have been developed to gather data regarding women’s health for the study). Recommendations provided for The Ottawa Statement on the Ethical Design and Conduct of Cluster Randomized Trials were taken into account in the design of this trial [32].

## Discussion

There is an urgent need for approaches to achieve effective and sustainable scaling-up of beneficial interventions in developing counties and particularly in low-resource settings. This can only be accomplished by the engagement of the country’s health stakeholders at all levels. This project aims to achieve improvement in the quality of antenatal care in Mozambique through the implementation of a multifaceted intervention on three levels: policy level, organizational level and health care delivery level.

Coordination of such a complex intervention is expected to be challenging. Foreseen difficulties include: organizational and logistical difficulties in delivering the intervention, adjusting to government, political and policy changes, and maintaining timelines, as much as possible. However, the methodology proposed integrates all required components to achieve the objectives and we have the cooperation of a multidisciplinary team of internationally recognized researchers and implementers for this project.

The idea of using the registration book as the instrument for data collection is critically important. It represents the best source of data for the primary outcomes of this research study because it is endorsed at the national level, and is mandatory for all health providers to complete. Importantly, by using this instrument, data collection for the demonstration project will not additionally burden the health professionals. The Ministry of Health has given the authorization to use this instrument to analyze ANC practices in the context of this study.

The proposal to gather effective and efficient data on the availability of supplies and to use this information to avoid shortages or stock outs, represents an innovative approach emerging from the formative research. This activity will require not only data collection, but also the use of the data collected to avoid stock outs. This will help ensure that supply chain management and the allocation of resources is based on relevant data and adjusted to local needs.

Implementing the proposed activities will also require a change in accountability and behaviour of health care workers (mainly health care providers) since participating in this kind of research requires an accurate and standardized registration of all activities involved in the health care process. We expect this change in “habits” will contribute to obtaining reliable health indicators, not only related to research issues, but also to health care outcomes derived from the new health care model.

At policy level, the results of this study may suggest a need for revision of the existing supply chain management system. Given that supply chain management is a major challenge for many low-resource countries like Mozambique, we envisage that important lessons with application to Mozambique and to other similar settings on how to improve the supply chain will be drawn from this study.

## Competing interests

The authors declare that they have no competing interests.

## Authors’ contributions

LC, MM, APB, JR, EB, MT and FA conceived the study and developed the first protocol. AA wrote the manuscript in collaboration with MLC and APB. LC, MM, APB, JR, EB, AA, MC, MLC, AC, BC, TD, DG, AMG, CRM, AM, MHN, NBO, MW, MT, FA participated in the design of the study and contributed to the writing and revising of the study protocol and manuscript. LC, MM, APB, JR, EB, AA, MC, MLC, AC, BC, TD, DG, AMG, CRM, AM, MHN, NBO, MW, MT, FA approved the final manuscript.

## Pre-publication history

The pre-publication history for this paper can be accessed here:

http://www.biomedcentral.com/1472-6963/14/228/prepub
